# Fabrication of hybrid nanocomposite scaffolds by incorporating ligand-free hydroxyapatite nanoparticles into biodegradable polymer scaffolds and release studies

**DOI:** 10.3762/bjnano.6.227

**Published:** 2015-11-25

**Authors:** Balazs Farkas, Marina Rodio, Ilaria Romano, Alberto Diaspro, Romuald Intartaglia, Szabolcs Beke

**Affiliations:** 1 Department of Nanophysics, Istituto Italiano di Tecnologia (IIT), Via Morego 30, 16163 Genova, Italy

**Keywords:** biodegradable scaffolds, biodegradation, hydroxyapatite, laser ablation in liquid, stereolithography

## Abstract

We report on the optical fabrication approach of preparing free-standing composite thin films of hydroxyapatite (HA) and biodegradable polymers by combining pulsed laser ablation in liquid and mask-projection excimer laser stereolithography (MPExSL). Ligand-free HA nanoparticles were prepared by ultrafast laser ablation of a HA target in a solvent, and then the nanoparticles were dispersed into the liquid polymer resin prior to the photocuring process using MPExSL. The resin is poly(propylene fumarate) (PPF), a photo-polymerizable, biodegradable material. The polymer is blended with diethyl fumarate in 7:3 w/w to adjust the resin viscosity. The evaluation of the structural and mechanical properties of the fabricated hybrid thin film was performed by means of SEM and nanoindentation, respectively, while the chemical and degradation studies were conducted through thermogravimetric analysis, and FTIR. The photocuring efficiency was found to be dependent on the nanoparticle concentration. The MPExSL process yielded PPF thin films with a stable and homogenous dispersion of the embedded HA nanoparticles. Here, it was not possible to tune the stiffness and hardness of the scaffolds by varying the laser parameters, although this was observed for regular PPF scaffolds. Finally, the gradual release of the hydroxyapatite nanoparticles over thin film biodegradation is reported.

## Introduction

Interfaces between osteochondral prosthetics and the surrounding bone tissue are of great importance with regard to the promotion and enhancement of biological fixation (firm bonding of the implant to the host bone by on-growth or ingrowth). Hydroxyapatite (HA) nanoparticles (NPs) are one of the most commonly used materials in osteochondral tissue engineering, since they bear chemical similarity to the mineral constituent of human bones, are bioactive and can be fairly easily bioconjugated [1]. HA NPs can enhance cell proliferation in bone tissue regeneration [2].

Tissue engineering is an interdisciplinary field that combines the principles of life sciences and engineering to improve tissue growth and functions. Whenever the need arises for a certain type of scaffold to be produced, all these fields have to be utilized together to get an appropriate solution.

HA is an essential ingredient of normal bone and teeth and is widely used for bone tissue regeneration. Given the high degree of chemical similarity between synthetic HA and the natural bone mineral, a large number of studies have introduced synthetic HA as bone replacement material for biomedical applications [3–4]. The benefits of synthetic HA, most notably its biocompatibility, slow biodegradability and good osteoconductive and osteoinductive capabilities [5–6], made it a platform for large-scale biomedical applications, such as controlled drug release and bone tissue engineering materials [7–8].

Lee et al. [9] reported on cellular responses to crosslinkable poly(propylene fumarate)/hydroxyapatite nanocomposites and showed that these nanocomposites are beneficial for hard tissue replacement due to the excellent mechanical strength and osteoconductivity. They used commercially available HA NPs, whereas we prepared HA NPs by PLAL and controlled the size by this method. They demonstrated also that the addition of HA enhanced hydrophilicity and serum protein adsorption, and as a result, this increased pre-osteoblast cell attachment, spreading, and proliferation after four days of culture.

Different technical routes have been explored for the synthesis of HA NPs, including mechanochemical synthesis [10], combustion preparation [11] and various wet chemistry techniques [12–13]. However, these routes have drawbacks regarding the synthesis attributed to the use of hazardous surfactants that are not suitable for biomedical applications [14].

Pulsed laser ablation of solid targets in liquids (PLAL) for the production of highly pure nanoparticles, has attracted research over the last decade mainly because of the simplicity of the method [15–18]. The main advantages of the technique include the green synthesis without chemical agents, a high production yield [17], the possibility to work under ambient conditions, and the versatility that allows for in situ manipulations [16].

Earlier, we had reported on the incorporation of Au NPs [19] and titanate nanotubes (TNTs) in and on the scaffolds [20] to develop poly(propylene fumarate)/diethyl fumarate (PPF:DEF) scaffolds for specific biomedical applications. We also proved that these scaffolds did not cause immune rejection [21]. A constant release of HA NPs during scaffold degradation may vastly improve the healing process. Also, certain tuning capabilities emerge with PPF:DEF resins when fabrication parameters are changed [22–23]: The stiffness/Young modulus of our rapid prototyping-fabricated scaffolds can be adjusted over a range of four orders of magnitude without any implied modifications concerning the chemical composition of the resin itself.

In this study, we present the combination of two laser methods (PLA and MPExSL) to incorporate HA NPs into biodegradable polymer resin. We also present HA NPs release studies.

## Results and Discussion

### Materials characterization

In order to evaluate the effects of nanoparticle incorporation, the cured resins have been characterized through nanoindentation, thermogravimetric analysis (TGA), profilometry, and FTIR measurements similar to a previous work performed on Au NPs embedded in the same polymer matrix [19]. Shortly, 2D samples were made to measure the changes of penetration depth (and thus, the layer thickness), while five layer, non-porous circular scaffolds with 2 mm diameter were prepared for nanoindentations. For TGA and FTIR, the same 20 layer, 5 mm diameter non-porous samples were used. For all 3D scaffolds, the layer thickness was adjusted to 100 µm. Most results were then compared to our previously acquired data.

**Figure 1:**
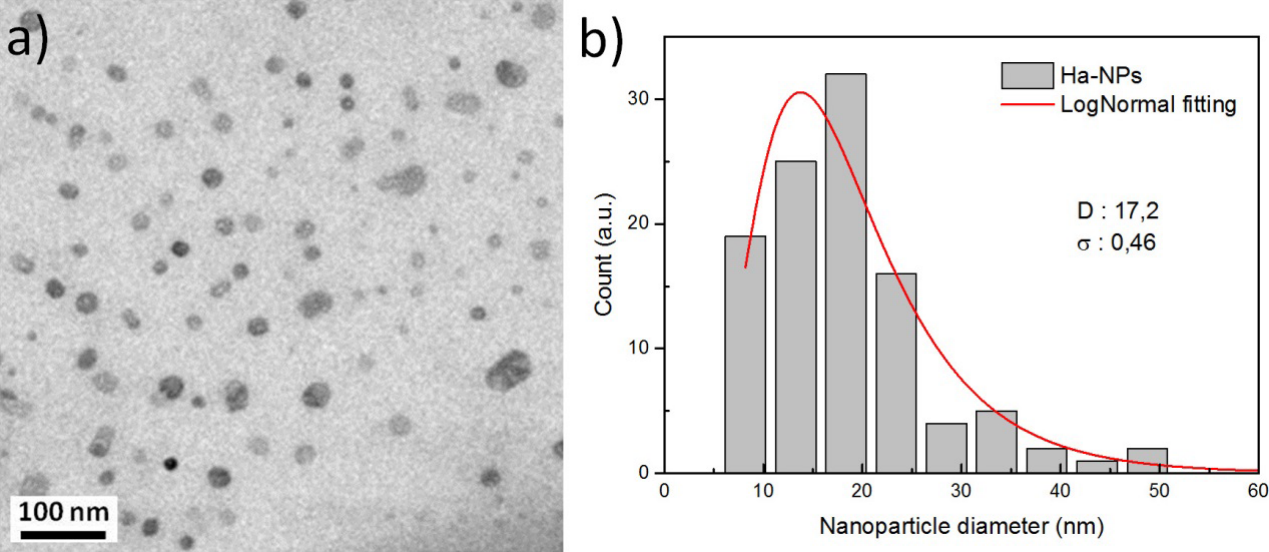
TEM image of hydroxyapatite colloidal solution prepared by UV laser ablation of hydroxyapatite target immersed in ethanol solution. b) Size distribution histogram of the colloidal solution revealing the mean size around 17 nm. The particle size distribution is obtained by using the ImageJ software. D and σ correspond to the mean size and the corresponding standard deviation obtained from a log-normal fitting, respectively.

Figure 1 shows a TEM image of hydroxyapatite colloidal solution (Figure 1a) prepared by UV laser ablation of a hydroxyapatite target in ethanol solution, and the corresponding size distribution histogram of the HA NPs (Figure 1b). The mean size of HA NPS was found to be around 17.2 nm.

**Figure 2:**
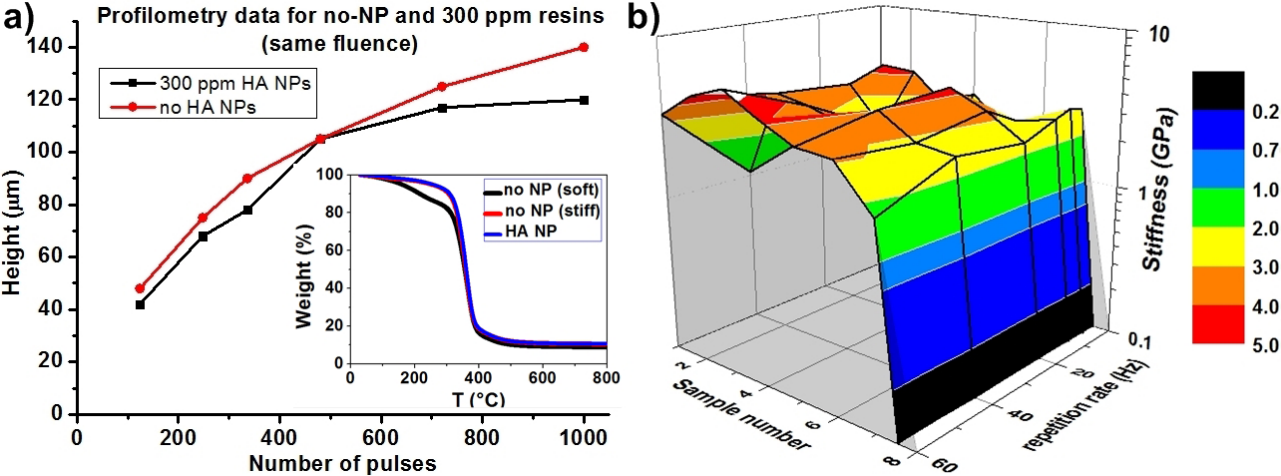
a) Profilometry data shows no disparity between the non-NP samples and the 300 ppm HA samples; inset presents TGA which shows that the HA samples exhibit the same behavior as the „high stiffness” no-NP samples; b) nanoindentation presents the lacking tuning capability of the HA samples.

Since the particles were already dispersed in ethanol, no surface modifications were needed to achieve direct compatibility between the particles and the PPF resin. As seen in Figure 2a, the added nanoparticles only barely affected the penetration depth of the light from the XeCl excimer laser (308 nm) even with the highest particle concentration. On the other hand, TGA (Figure 2a inset) and nanoindentation (Figure 2b) measurements show that the stiffness of the fabricated samples can be considered identical, independent of the concentration of the nanoparticles, as well as the laser parameters. Also, the samples (samples 1–6) fabricated with three different HA concentrations (50, 100 and 300 ppm) and two different fluences (10 and 30 mJ/cm^2^) show the same behavior as the ones fabricated without nanoparticles at 10 mJ/cm^2^ (which corresponds to the highest stiffness achieved before, sample 7), independently of the fluence. When the highest fluence (30 mJ/cm^2^) is used on the non-NP sample, it leads to a sharp drop in the stiffness (sample 8), just as expected from our previous results [23].

**Figure 3:**
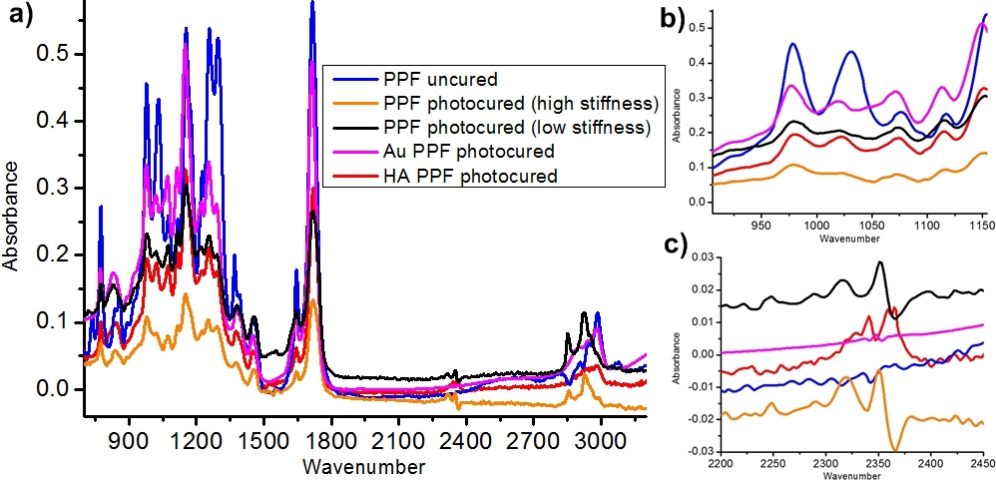
a) Comparison of the FTIR spectra received for HA samples and samples from previous studies [19,23]. All HA show the same FTIR, following closely the „high stiffness” samples fabricated without any nanoparticles. The sole differences that can be found is where the main peak of the pure HA NPs is and where the three carbonate peaks arise (HA reacting with environmental CO_2_); b) close-up of peaks 1020 and 980 cm^−1^ and c) is a close up of peaks at 2340, 2360 and 2365 cm^−1^.

FTIR (Figure 3a) shows no disparities in the chemical composition of the HA samples compared to the „high stiffness” pristine samples, apart from a slight difference in the ratio of the peak height of the peaks 1020 and 980 (Figure 3b). Three new peaks also emerge at 2340, 2360 and 2365 cm^−1^ (Figure 3c). These latter three are obvious markers of the carbonates formed by the reaction between the Ha NPs and the CO_2_ in the air during the laser processing. The slight change in the peak ratio at 1060 cm^−1^ is supposedly due to the HA NPs having a prominent peak there.

From these measurements, we can claim that HA NPs do not react chemically with the resin during the photocuring process. They do not absorb either, but act like scattering points for the incoming UV irradiation and therefore increase the photocuring efficiency. This scattering effect also reduces any kind of tuning capability that we observed before on non-NP and Au NP resins, resulting in samples with uniformly high stiffness. In other words, the previously presented possibility to tune the stiffness is not observed.

### Nanoparticle resin

**Figure 4:**
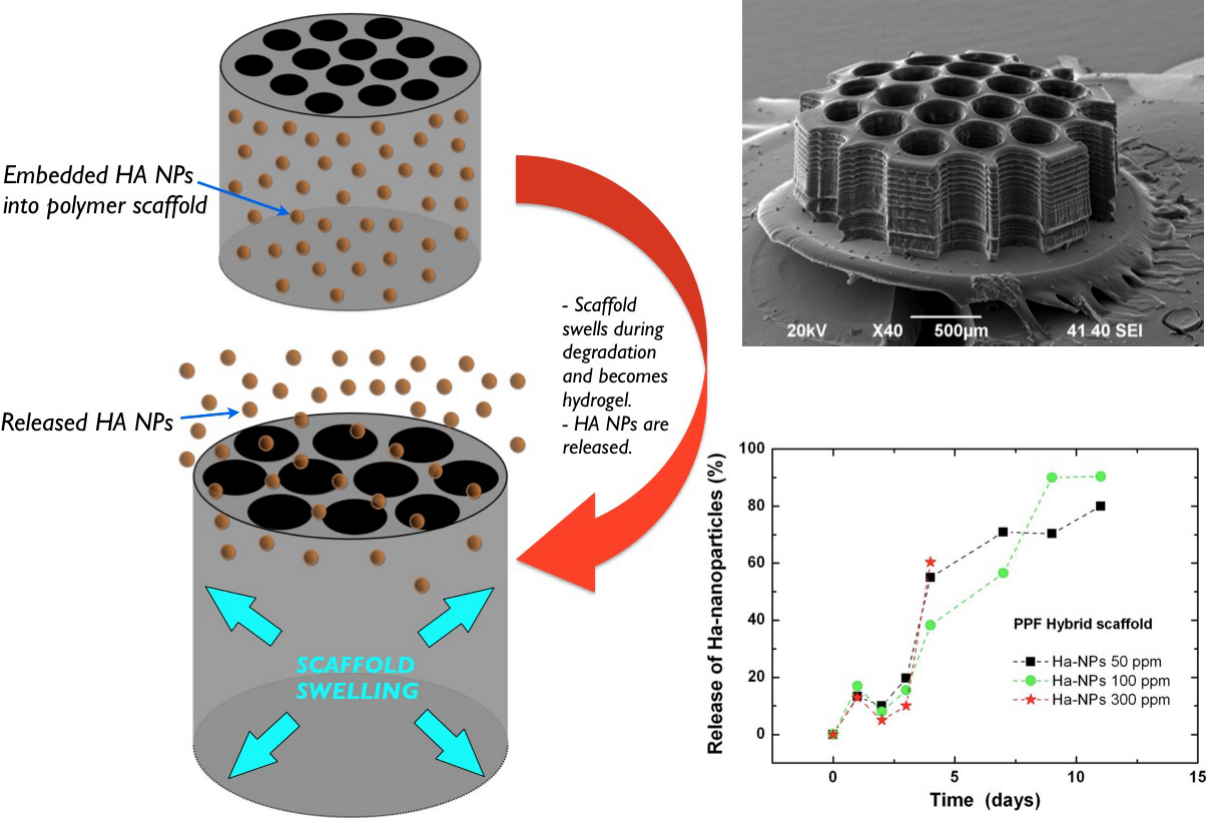
Release and degradation of HA NP-incorporated polymer scaffolds are presented during its degradation in DMEM. The scaffold becomes hydrogel, swells and releases the HA NPs. The plot shows the released fraction in percent as a function of the time, estimated by inductively coupled spectrometry (ICP-EOS).

Figure 4 illustrates the degradation, the scaffold swelling, and HA NPs release during degradation in Dulbecco's Modified Eagle's Medium (DMEM). In order to test the release of HA NPs from the bulk scaffolds, 20 layer of 3 mm diameter porous (400 μm pore size) scaffolds were fabricated from HA NPs–PPF resins containing HA NPs with different concentrations of 50, 100 and 300 ppm (Figure 4, inset). The scaffolds were immersed in 100 μL of DMEM solution for 4 weeks. The degradation medium was extracted daily, then 3 times, 2 times and once a week. The release profile of Ha NPs during the PPF scaffold degradation as a function of time was estimated by inductively coupled plasma optical emission spectrometry measurements (ICP-OES, ICAP 6300 duo ThermoScientific). Every time a volume of 25 μL was taken for ICP-EOS analysis and replaced by the same volume of fresh DMEM solution buffer.

Results have shown a stable increase in the amount of particles in the DMEM for the first two weeks. When normalized (to percentage and considering the diluting effect of the replaced medium), all concentrations have shown the exact same release mechanism: a fast ramp up for the first two weeks, followed by saturation at 80–90% released HA NPs. This release mechanism fits well to our empirical observations concerning the degradation of high stiffness PPF scaffolds in DMEM: the material becomes a hydrogel during the first two weeks, then disperses into the medium in the next 4–6 weeks. Such degradation obviously leads to a ramp up in the release mechanism due to the hydrogel transition phase. Of note, the experiment had to be cancelled after 2 weeks due to contamination: the vials became infected by fungi, gradually digesting the nanoparticles (data not shown).

## Conclusion

We presented the combination of two laser processing methods (PLA and MPExSL) to incorporate hydroxyapatite nanoparticles (HA NPs) into a biodegradable polymer resin. Ligand-free production of NPs can be considered a green route of NP synthesis that is beneficial for biological applications. HA NP release test was performed and showed that a controlled release of HA NPs is feasible and highly favorable since the HA is widely utilized along with prosthetics these days and can only supply the particles for a few days before the pure, sprayed-up HA layer completely disperses into the surrounding tissue. With the PPF-HA NP resin, a stable dosing can be achieved for the most crucial first two weeks of the healing process.

## Experimental

### Poly(propylene fumarate)

Poly(propylene fumarate) (PPF) has been chosen as the carrier for the HA nanoparticles following our previous success with gold nanoparticles (Au NPs) [19]. PPF itself is a versatile synthetic biopolymer, a biodegradable and photocurable material. The photocrosslinking efficiency is usually adjusted by the added photoinitiator (PI) concentration („chemical tuning”), though we recently demonstrated that great stiffness tuning (over four orders of magnitude) could be achieved by changing various fabrication/laser parameters as well („physical tuning”) [23] with pristine resins as well as the Au NP ones. The obvious advantage of the chemical tuning over physical tuning is reliability, while physical tuning can be conducted in situ, leading to a highly versatile, albeit hard to evaluate, fabrication procedure. With the physical tuning, it is also possible to achieve complex scaffold structures, for instance, mimicking composite materials.

The PPF used in this work is not commercially available. The synthesis is reported elsewhere [22]. The PI phenylbis(2,4,6-trimethylbenzoyl)phosphine oxide (BAPO) was dissolved in purified PPF and in the PPF:DEF (7:3 w/w) blend. Diethyl fumarate (DEF) is applied as diluent to reduce the resin viscosity as needed for the proper resin recast for MPExSL.

### MPExSL

Mask-projected excimer laser stereolithography (MPExSL) is a rapid prototyping stereolithography method, relying on a layer-by-layer building-up process where one layer is fabricated by image projection using pulsed excimer laser radiation. The method is explained in detail in [22].

Briefly, the method goes as follows: the output beam of a XeCl excimer laser (at 308 nm wavelength) is coupled to a customized mask projection optical system with a telescope and a motorized variable attenuator. The system also includes a motorized mask holder (which can house five masks at the same time) that selects the image to be projected on the resin surface, adding great control for the scaffold internal architecture for each layer. The outer shape of the scaffold (e.g., the scaffold diameter in the horizontal plane) can be further adapted by means of an iris placed right in front of the mask holder [22]. Above the optical setup, a CCD camera is housed to on-line image the resin surface and in situ monitor the fabrication process.

During the stereolithography process, the photocurable resin is filled in a cup supported by a multi-axis motorized stage. The proper position of the resin surface to be exposed by the laser is achieved by moving the sample with an XYZ stage, while a fourth motorized stage (W) controls the vertical position of the scaffold-holding platform immersed in the resin container cup. After one layer is photocured the W stage moves downwards into the resin and allows the recast of a fresh liquid resin layer on top of the previously cured layer. The applied light dose determines the actual photocured depth, i.e., layer thickness, while the magnitude of the downward step of the W stage determines the overlap between two adjacent layers.

### Hydroxyapatite nanocomposite synthesis by using two laser-processing methods

**Figure 5:**
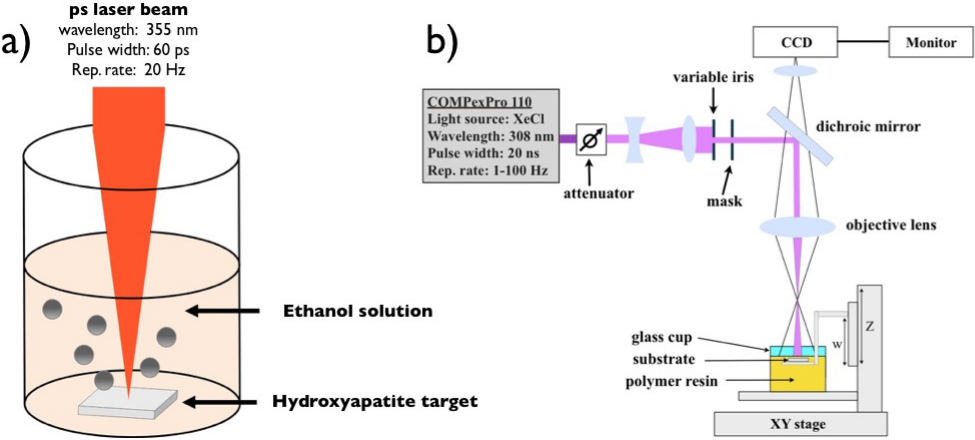
a) Illustration of the ultrafast laser-assisted method for the synthesis of HA colloidal solution and b) MPExSL setup for the scaffold production with incorporated HA NPs.

Figure 5 presents the experimental setups of the two laser-assisted methods applied for HA/PPF nanocomposite production.

HA NPs were prepared by UV laser ablation of a HA target placed in ethanol solution using a picosecond laser system (continuum leopard) delivering 60 ps laser pulses with a wavelength of 355 nm at a repetition rate of 20 Hz. The synthesis was performed following the previous work [15]. The laser pulse energy was controlled with a variable attenuator and fixed at 42 mJ. A lens with a focal length of 250 mm and a focal position of −10 cm with respect to the target surface was used. The target material (hydroxyapatite from CELLYARDTM pellet, PENTAX), in the form of a cylinder with a diameter of 5 mm and a thickness of 2 mm, was placed on the bottom of a quartz cuvette (dimension 10 × 10 × 30 mm^3^) with 2 mL of ethanol solution. The target was placed on a motorized stage (T-cube DC Servo controller, Thor labs) that moved at a constant speed of 1 mm/s in a spiral with an outer radius of 1 mm. The irradiation time was fixed at 120 min. Particle size distribution was evaluated by TEM.

The HA NP/ethanol colloidal solution was added to the PPF:DEF during resin production: The colloidal solution was mixed with DEF, then added to the PPF in 7:3 w/w followed by 1% photoinitiator. In the end, the resin was stirred for 24 h under a hood to evaporate all ethanol. Various concentrations of HA NP resins were made from colloidal with concentrations of 20, 50, 100 and 300 ppm. Each solution was 5 mL.

### Characterizations

Inductively coupled plasma optical emission spectrometry measurement (ICP-OES, ICAP 6300 duo thermo scientific) was used to determine the quantity of Ha colloidal solution. For this measurement, 25 µL solution of NPs colloidal solution was introduced in aqua regia, and after overnight acid digestion the final volume was adjusted with Milli-Q water to 25 mL. The dilution factor is kept into consideration while determining the final concentration.

Height measurements were carried out by a Veeco Dektak 150 profiler with 2 mg of load on the tip.

Stiffness measurements were performed by using a Micro Materials Ltd. NanoTest. The tests were conducted by applying a Berkovich tip with a maximum load of 0.6 mN, a dwell time at maximum load of 30 s, loading and unloading periods of 30 and 15 s, respectively. Every sample has been measured at 16 different points (in a matrix of 4 × 4, the distance between measurement points was 50 μm). Young’s modulus was calculated through the Oliver and Pharr method each time. The stiffness of the whole sample was acquired eventually by calculating the mean value of these aforementioned 16 points.

Thermogravimetric analysis (TGA) was conducted in a TGA Q500 from TA Instruments. The sample was placed in a platinum pan with an equilibrating step at 30 °C. The annealing went to 800 °C with a 10 °C/min rate. The nitrogen flow was 50 mL/min.

Transmission electron microscopy (TEM) was performed with a JEOL Jem1011 microscope working at an acceleration voltage of 100 kV. Samples were prepared by dropping the colloidal solution directly onto a carbon-coated 300 mesh copper grid and allowing the ethanol solution to evaporate under room temperature and pressure.

FTIR spectroscopy analysis was performed by using the Bruker Vertex 80V infrared spectrometer. HA-NPs scaffold samples were analyzed in transmission mode in the range of 600–4000 cm^−1^.
